# Polycyclic aromatic hydrocarbons and stable isotopes of carbon and nitrogen in Baltic Sea blue mussels: Time series data 1981–2016

**DOI:** 10.1016/j.dib.2021.106777

**Published:** 2021-01-18

**Authors:** Agnes M.L. Karlson, Suzanne Faxneld

**Affiliations:** a Department of Ecology, Environment and Plant Science, Stockholm University, SE-106 91 Stockholm, Sweden; b Department of Environmental Research and Monitoring, Swedish Museum of Natural History, P.O. 50007, SE-104 05 Stockholm, Sweden

**Keywords:** Contaminant monitoring, Long-term data, Isotope baseline, PAH, Biogeochemical tracer, Pollution

## Abstract

Blue mussels are a target species in contaminant monitoring regarding Polycyclic Aromatic Hydrocarbons (PAHs) in biota, and also used as an isotope baseline for trophic position assessment in other biota. The latter is crucial for calculating biomagnification potential of environmental contaminants. This data set comprises long-term time series of PAHs (15 individual substances) in Baltic Sea blue mussels (*Mytilus trossulus edulis*) from Kvädöfjärden (collected from a depth of 5–10 m), a reference area along the Swedish coast in the Baltic Proper from 25 years during 1987–2016, and of stable isotopes in five individuals (2 cm mussels) per year during the time period 1981–2017. The data has been co-analysed with environmental (oceanographic) data in “The importance of adjusting contaminant concentrations using environmental data: a retrospective study of 25 years data in Baltic blue mussels” published in: Science of the Total Environment (https://doi.org/10.1016/j.scitotenv.2020.143913).

## Specifications Table

**Table:** 

Subject	Environmental science: Ecology and Environmental Chemistry
Specific subject area	Environmental and contaminant monitoring
Type of data	Table(s): Table S1: 15 PAHs measured in one pooled sample per year 1987–2016 Table S2: Stable carbon and nitrogen isotopes measured in individuals (*n* = 5 per year) 1981–2017
How data were acquired	Collection of blue mussels in the field are described under “Parameters for data collection” and included in the Swedish Contaminant monitoring program [1]. After dissection of shells, the soft tissue of pooled mussels samples were analysed for PAHs (15 individual substances), and individual mussels with a shell length of 2 cm were dried and retrospectively analysed for stable isotopes. Instruments: PAH analyses were performed using HPLC type Agilent 1290, column Pursuit PAH 100* 3 mm, analysed at IVL, the Swedish Environmental Research Institute, Gothenburg, Sweden. Stable isotope analyses using EA-IRMS, elemental analyser PDZ Europa 20–20 isotope ratio mass spectrometer (Sercon Ltd., Cheshire, UK), at UC-Davis Stable Isotope Facility, CA, US.
Data format	Raw data
Parameters for data collection	The mussels were collected yearly from 1987 until 2017, frozen directly after collection, prepared for the analysis as described below, analysed for PAHs (15 compounds) and stable isotopes and elemental composition of carbon and nitrogen as described below.
Description of data collection	PAH data (naphthalene, acenaphthene, fluorene, phenanthrene, anthracene, fluoranthene, pyrene, benzo(a)anthracene, chrysene, benzo(b)fluoranthene, benzo(k)fluoranthene, benzo(a)pyrene, benzo(g,h,i)perylene, dibenzo(a,h)anthracene, indeno(1,2,3-c,d)pyrene) in Table 1 originate from the Swedish Contaminant monitoring (see “How data were acquired”). Stable carbon and nitrogen isotope and elemental data in Table 2 originate from retrospective analyses of leftover mussels not analysed for contaminants in the monitoring program but kept frozen in the Environmental Specimen Bank (see “How data were acquired”).
Data source location	Institution: Swedish Museum of Natural History and Stockholm University City/Town/Region: Stockholm Country: Sweden Latitude and longitude for collected samples/data: N 58,033645°, E16,764972° (Kvädöfjärden, Baltic Sea)
Data accessibility	The data is with the article as supplementary files (Table 1 and Table 2) and also uploaded on the Swedish publicly available site at the Geological Survey of Sweden, www.sgu.se, as required by the Swedish Environmental Protection Agency, but instructions on how to access data is only in Swedish: https://www.sgu.se/produkter/geologiska-data/oppna-data/miljo-oppna-data/marina-data-i-atomfloden/
Related research article	Ek C, Faxneld S, Nyberg E, Rolff C, Karlson AML. 2021. The importance of adjusting contaminant concentrations using environmental data: a retrospective study of 25 years data in Baltic blue mussels. Science of Total Environment. https://doi.org/10.1016/j.scitotenv.2020.143913

## Value of the Data

• Blue mussels are a target species in contaminant monitoring regarding Polycyclic Aromatic Hydrocarbons (PAHs) in biota as required by national and EU legislation [2], [3], [4], [5]. Blue mussels are also recommended as a nitrogen isotope baseline for trophic position assessment in other biota. The data in this article are important for understanding long-term changes in both PAH concentrations and stable isotopes at the base of the food web.
• Long-term data is key for assessing the efficiency of measures taken to reduce human pressures, such as eutrophication or contaminant loading, on the environment. Long-term time series from monitoring is often the most important source of information for understanding marine ecosystem dynamics, since long-term ecological research programs only rarely exist globally. Hence, both contract holders for monitoring programmes and researchers will greatly benefit from long, uninterrupted time series data.
• Long-term data on contaminants provide baselines for anthropogenic pressure in the environment and is needed for assessing ecosystem health. Stable isotopes of carbon and nitrogen can be used to infer large scale biogeochemical changes in the environment [6], but also to study food web interactions. Mussels are particularly suitable as isotope baselines since they are sessile and long-lived [7]. For this reason they are used when estimating trophic position in higher consumer and hence calculations of biomagnification potential of contaminants [8]; this time series of stable isotopes in a baseline organism now allow for retrospective analyses of trophic position over time in fish from this reference site.

## 1 Data Description

Data in supplementary Table S1 ( excel sheet) contain annual rawdata on concentrations of the 15 individual PAHs in blue mussel soft tissue (ng g^−1^ shell-free dry weight) measured in a pool of mussels (>20 individuals) during 1987–2016 from station Kvädöfjärden, Baltic proper. Full name and abbreviation for each PAH is given in the specification table above and in the first row of Table 1. For acenaphthene, values are rarely found above the level of quantification (LOQ), values below this level are marked in grey and represent absolute values divided by the square root of 2.

Data in supplementary Table S2 contain individual (*n* = 5 per year) nitrogen and carbon stable isotope data (expressed in the delta notation, δ^15^N and δ^13^C (‰ deviations from an international standard) as well as the carbon: nitrogen elemental ration (weight: weight) during 1981–2017. Data is organized after the unique sample ID from the Environmental Specimen Bank, year collected, the δ^13^C values, δ^15^N values, C:N ratio and a column with comments (three outliers for δ^13^C values were identified).

## 2 Experimental Design, Materials and Methods

### 2.1 Sampling and laboratory measurements

Mussel collection was conducted annually during October-November from 5–10 m depth (bottom substrate clay and gravel) at exactly the same position every year (N 58,033645°, E 16,764972°) using coastal survey nets; mussels are bycatch from sampling of fish from this site and position. For the period 1981–2017 data is missing for six years (1983, 1992, 2000–2002, 2004). PAHs are measured since 1987 (hence the years 1992, 2000–2002 and 2004 are missing). Mussels were immediately frozen after collection and stored at −25 °C at the Environmental Specimen Bank at the Swedish Museum of Natural History, Stockholm Sweden.

During sample preparation prior to analyses in the laboratory, individual specimens were thawed and carefully opened using a metallic scalpel. The soft tissue (not including the adductor muscle) was removed and placed in a glass beaker. For each specimen maximum shell length was registered. One pooled sample (*n* = 20–75 individuals, depending on size of sampled mussels a particular year; within a year only similar sized individuals were used) was prepared each year and homogenised using an IKA T25 digital ULTRA TURRAX homogenizer. The homogenate was analysed for 15 PAHs at the Swedish Environmental Research Institute (IVL) as described below.

### 2.2 PAH analyses

The extraction and analysis of the samples were performed according to IVLs accredited method for PAH. The biota samples were spiked with recovery standard (2,2′-Binaphthyle), homogenised in acetone and extracted in an ultrasonic bath. The extract was safeguarded and the samples were extracted once more with acetone and twice with pentane/ether. The extracts were combined and the organic compounds were extracted to an organic phase by liquid/liquid extraction with water and pentane, and further concentrated under nitrogen. The samples were hydrolysed and pre-treatment procedures, such as fractionation of the organic compounds on silica, were performed as additional "clean-up" procedures. Laboratory blanks followed the same procedures as samples in the analytical work.

Determination of PAH components was carried out using a high performance liquid chromatograph (HPLC, type Agilent 1290, with a 3 μm C18-column (Pursuit PAH 100* 3 mm, Agilent). A linear gradient elution program was used, starting with acetonitrile/water 50:50 and ending with 100% acetonitrile (Rathburne HPLC-grade). A fluorescence detector (Agilent 1260) with a wavelength program optimised for each PAH was used for quantification. The peak heights were registered with a chromatographic system from Thermo (Chromeleon 7.0). The concentrations of 15 different PAH compounds were calculated by comparison to a certified standard, NIST, SRM 1647f (Priority Pollutant Polycyclic Aromatic Hydrocarbons in Acetonitrile).

All of the standards used (both the recovery standard and quantification standards) are certified with known purity and precision. Certified reference material (CRM) was run in parallel to the mussel samples and used to check the method performance (NIST, SRM 2974a, Organics in freeze-dried mussel tissue (*Mytilus edulis*). Concentrations are expressed as ng g^−1^ shell-free dry weight; a subsample of the mussel soft tissue homogenate was dried at 105 °C at 20 h.

### 2.3 Stable isotope analyses

Shells from thawed mussels from the Environmental Specimen Bank were dissected and subsamples of homogenized dried (60 °C for 48 h) mussel soft tissue (individual samples, *n* = 5 per year) were placed in tin cups (1 mg dry weight) and sent for stable isotope ratio analyses (^13^C:^12^C and ^15^N:^14^N) and elemental analyses (C, N content% of dry weight) at the UC Davis Stable Isotope Facility, US, through combustion using a PDZ Europa 20–20 isotope ratio mass spectrometer (Sercon Ltd., Cheshire, UK). Isotope data are expressed in the delta notations, δ^15^N and δ^13^C, which is ‰ deviations from an international standard (nitrogen in air for δ^15^N and Vienna Pee Dee Belemnite, VPDB for δ^13^C; the latter is nowdays back-calculated from a widely measured carbonate standard NBS-19). Reference materials measured every tenth sample are Bovine Liver, Glutamic Acid, enriched Alanine and nylon 6.

## Ethics Statement

Data originate from invertebrates which require no special permits for sampling in the Baltic Sea.

## CRediT Author Statement

**Agnes ML Karlson:** Conceptualization, Data curation, Writing- Original draft preparation; **Suzanne Faxneld**: Investigation, Reviewing and Editing.

## Declaration of Competing Interest

The authors declare that they have no known competing financial interests or personal relationships which have or could be perceived to have influenced the work reported in this article.

## Supplementary materials

Image, application 1

## Footnotes

Supplementary Tables 1 and 2 contain the data presented. Supplementary material associated with this article can be found in the online version at doi:10.1016/j.dib.2021.106777.
